# Memory training combined with 3D visuospatial stimulus improves cognitive performance in the elderly: pilot study

**DOI:** 10.1590/1980-57642020dn14-030010

**Published:** 2020

**Authors:** Mariana Medeiros Assed, Cristiana Castanho de Almeida Rocca, Yolanda Maria Garcia, Tatiana Cohab Khafif, Gabriel Okawa Belizario, Edgar Toschi-Dias, Antonio de Pádua Serafim

**Affiliations:** 1 Neuropsychology Unit, Institute and Department of Psychiatry, Faculdade de Medicina, Universidade de São Paulo, São Paulo, SP, Brazil.; 2 Department of Geriatrics, Faculdade de Medicina da Universidade de São Paulo, São Paulo, SP, Brazil.; 3 Health Psychology Program, Universidade Metodista São Paulo, São Bernardo do Campo, SP, Brazil.

**Keywords:** aged, memory training, memory, neuropsychology, rehabilitation, idoso, aprendizagem, memória, neuropsicologia, reabilitação

## Abstract

Studies suggest that the engagement of aged participants in cognitive stimulation programs can reduce expected cognitive decline associated with age.

**Objective:**

To evaluate the effects of memory training (MT) associated with three-dimensional multiple object tracking (3D-MOT) NeuroTracker (NT) in the elderly.

**Methods:**

Forty-four participants (>60 years of age) were recruited and randomly distributed into two groups: experimental (EG; n=22) and comparative (CG; n=22). Both groups performed 12 one-hour MT sessions, twice a week, consisting of specific computerized stimuli associated with teaching of mnemonic strategies; 10 minutes of NT was part only of the EG’s sessions. In pre- and post-training periods, both groups were evaluated using a sociodemographic questionnaire, neuropsychological assessment, as well as a specific measure offered by NT.

**Results:**

Both groups benefited from the MT and reported more positive feelings regarding their memory and quality of life. However, the EG obtained better results in tests consistent with the strategies trained and which involved attentional resources, reaction time, visual processing speed, episodic, semantic, subjective and working memory as well as aspects of social cognition.

**Conclusions:**

This study showed that the combination of MT and 3D-MOT contributed for a better cognitive performance in the EG. Thus, the results of the present study encourage further research and the development of combined cognitive interventions for the elderly population with and without cognitive deficits.

## RESUMO.

Estudos sugerem que o envolvimento de idosos em programas de estimulação cognitiva pode reduzir o declínio cognitivo esperado associado à idade.

**Objetivo:**

Avaliar os efeitos de um treinamento de memória (TM) associado a um estímulo visuoespacial tridimensional (3D-MOT) NeuroTracker (NT) em idosos.

**Método:**

Quarenta e quatro participantes (>60 anos) foram recrutados e distribuídos aleatoriamente em dois grupos: experimental (GE; n=22) e comparativo (GC; n=22). Ambos os grupos realizaram 12 sessões do TM de uma hora, duas vezes por semana, consistindo em estímulos computadorizados específicos associados ao ensino de estratégias mnemônicas; apenas nas sessões do GE foram utilizados 10 minutos com o NT. Nos períodos pré- e pós-treinamento, ambos os grupos foram avaliados por meio de questionário sociodemográfico, avaliação neuropsicológica e medidas cognitivas específicas do NT.

**Resultados:**

Ambos os grupos se beneficiaram do TM e relataram sentimentos mais positivos em relação à memória e à qualidade de vida. No entanto, o GE obteve melhores resultados em testes consistentes com as estratégias treinadas e que envolviam recursos atencionais, tempo de reação, velocidade de processamento visual, memória episódica, semântica, subjetiva e de trabalho, além de aspectos da cognição social.

**Conclusões:**

Este estudo mostrou que a combinação do TM e 3D-MOT contribuiu para um melhor desempenho cognitivo no GE. Dessa forma, os resultados do presente estudo incentivam novas pesquisas e o desenvolvimento de intervenções cognitivas combinadas para a população idosa com e sem déficits cognitivos.

Cognitive decline is an important cause for concern in aging populations; as it encompasses greater possibility of dementia, which causes dependency and incapacity in the physical, psychological, social, familiar, and economic spheres.^1^ However, studies suggest that the engagement of elderly participants in cognitive stimulation programs can reduce expected cognitive decline associated with age, as well as the decline recorded in clinical cases.^2^ ^,^ ^3^ ^,^ ^4^ ^,^ ^5^ ^,^ ^6^ Cognitive stimulation is associated with the synaptic plasticity that appears in normal learning and recovery processes and is directly related to experience.^7^ Therefore, it is an essential intervention for cutting losses in populations with deficits, acting as a preventive tool capable of potentializing cognitive functions such as memory.^3^ ^,^ ^4^ ^,^ ^5^ ^,^ ^6^ Studies focusing on the magnitude and maintenance of the benefits related to cognitive interventions indicate that plasticity is considerable in healthy individuals throughout life.^8^ Curiously, elderly individuals participating in cognitive training tend to obtain positive results in favor of dysfunctional connectivity.^9^ Bender et al.,^10^ showed the need to create a hierarchy of trained functions, in which positive results are directly related to the training of basic attentional functions first and, then, high-level control functions. This happens because attention includes a range of cognitive functions, involving focused, sustained and divided attention as well as information processing speed. Divided attention establishes simultaneous engagement in two cognitive tasks and demands good memory. It is possible that aged participants present difficulty with such activity and, consequently, express low performance in tasks that demand the use of different types of memories such as associative, recognition, and short term.^10^

In a computerized cognitive training meta-analysis, the authors did not find such robust results.^11^ Considering 52 articles with a total of 4,885 participants, results showed that the intervention designs differed considerably between studies. Both the experimental (EG) and comparative groups (CG) presented small to moderate effects in verbal and non-verbal memory, working memory, processing speed, and visuospatial abilities.

No significant effects were found for executive functions and attention. The authors concluded that this modality of training is modestly effective in improving cognitive performance in healthy elderly participants. However, the efficacy varies between cognitive domains and is largely determined by the choice of methodology. It is possible that the variation in study designs does contribute to these less significant results.

Considering the scarce evidence found in the literature, we have developed a pilot intervention program based on memory training (MT) associated with the Three-Dimensional Multiple Object Tracking (3D-MOT) NeuroTracker (NT), which is previously unseen in the literature on the elderly population, except for the publication of a case report in our group.^12^ The choice for NT was made based on the idea of hierarchical trained functions. In addition, NT was developed to train peripheral vision and is mainly associated with divided attention stimulation, operational memory, and speed processing.^13^ ^,^ ^14^ Note however, that studies utilizing this instrument have been mainly developed for athletes and showed improvement in performance related to processing, information learning as well as improvement in performance in sports.^15^ ^,^ ^16^ Studies also show improvement in the executive functioning of young adults who are not athletes^14^ and in the working memory of military officers.^17^

Based on these findings, the current study aimed to explore the effectiveness of MT combined with NT in the elderly population without cognitive complaints, taking into account the aging rate and the need to structure interventions that can address the typical cognitive changes of this population. Our hypothesis is that the associated use of MT and NT is more effective than when applied individually.

## METHODS

### Study design and sample

Forty-four healthy aged participants (>60 years old) without cognitive complaints were recruited to participate in this pilot study and randomly distributed into two groups. The sample consisted of the EG, with 22 participants (n=22) who underwent MT with a 3D-MOT software, and the CG (n=22), whose participants underwent only MT. Inclusion criteria were: being aged 60 years old or older; of both gender, a minimum of four years of formal education, physical ability to participate in all training sessions, as well as no history of depression or other psychiatric and/or neurological disorders.

### Instruments

- *Sociodemographic questionnaire*: created by this research group in order to collect information about age, gender, and education.
- *Mini Mental State Examination (MMSE):* a widely used test of cognitive function among the elderly; it includes tests of orientation, attention, memory, language, and visual-spatial skills.^18^
- *Wechsler Abbreviated Scale of Intelligence (WASI)*: Vocabulary and Matrix Reasoning Sub-tests,^19^ used to calculate the estimated intelligence quotient (IQ).
- *Verbal Fluency Test:* involves generating as many words as possible in a fixed period of time. The participant must evoke words that begin with the letters F, A and S and under the “animal” category.^20^
- *The Memory Check-up* **:** composed of five tests that measure: *i*) Simple Reaction Time (SRT), which represents the reaction time to a visual stimulus (test 1); *ii*) Choice Reaction Time (CRT), which represents the speed of a motor response to a visual stimulus after decision-making (test 2); *iii*) Episodic Memory (EM), which evaluates the capacity to acquire, store, and retrieve previously observed stimuli, as well as memories of details and emotions experienced at specific times and places (test 3); *iv*) Working Memory (WM), which tests the capacity for short-term storage of information, useful for immediate reasoning (test 4); and *v*) Verbal Memory (VM), which measures the capacity to acquire, store, and retrieve language-related information (test 5). The results of the reaction time and working memory tests were collected in milliseconds, the episodic and verbal tests in percentage of correct answers, and a composite score was calculated for a more structured comparative result.
- *NeuroTracker*: used as a computerized attentional stimulus for 3D visuospatial perception (3D-MOT) and for assessment of sustained and focused attention. For this evaluation, the scores were analyzed in two ways: (NT-H) or *Skill rating* - the participants’ scores based on the abilities attained in the exercise - according to how skilled they are able to become in the exercise; and (NT-P) or *Scores*, which is a score derived from the average relative to the visual processing speed and the difficulty level each participant is able to reach.^13^ ^,^ ^14^ ^,^ ^15^ ^,^ ^16^ ^,^ ^17^

### Procedures

#### Initial and final evaluation

**Figure 1.**
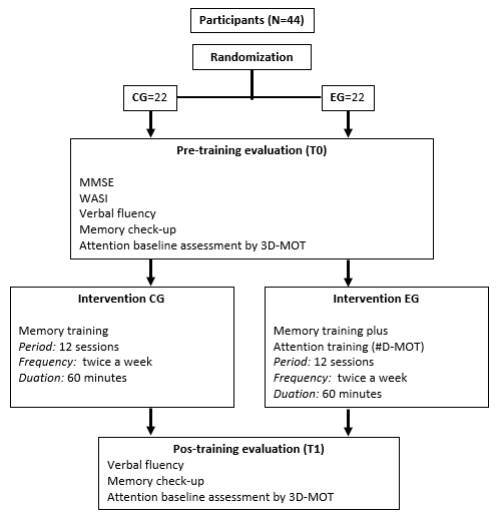
Sample flow.

The MMSE for cognitive impairment screening was used only in the pre-training phase. The remaining instruments were used to assess the possible training effects. Therefore, all participants were individually assessed before and after the 12 training sessions (Figure 1).

#### Cognitive training

For the MT, 12 sessions of 60 minutes were organized for groups with a maximum of 10 participants. Specific computerized stimuli were used, associated with the conscious teaching of mnemonic strategies - verbal and visual (rhyming memories and images); expanded/spaced repetition (involving the presentation of the content to be remembered followed by immediate testing and gradual retention intervals); errorless learning (technique to avoid making mistakes as people learn new information); and vanishing cues (stimuli are presented and gradually withdrawn). Participants are taught how to use the acronym PQRST, which stands for: *Preview* (establish the general theme of the text), *Question* (formulate main questions about the text), *Read* (read carefully, thinking about the questions), *State* (summarize the main information), and *Test* (test your knowledge).^21^ A computer equipped with 3D-MOT (NT) was used for specific attention stimulation activity in the EG, where focused and sustained attention exercises alternated for a period of 10 minutes each, during every session.

**Figure 2.**
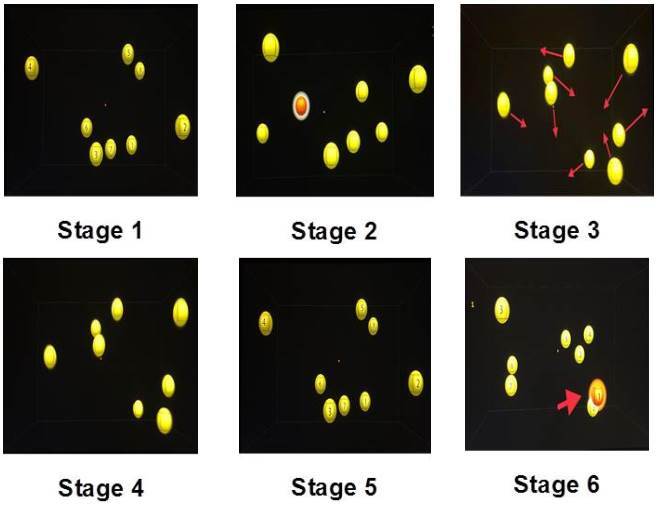
Presentations of the 3D stimulus.

The NT TMCORE program distributed by CogniSensAtletics Inc. was used to present the basic evaluation of the 3D-MOT in Figure 1. In stage 5, if participants were able to correctly identify the indexed sphere, the speed and difficulty levels increased in the next attempt and decreased in case of any mistakes. This mechanism happens throughout the whole extent of the activity. In each correct attempt, a star is used as a visual reward feedback. All forty-four participants were given procedural instructions, a computerized attention exercise and MT; however only the EG had NT sessions before the MT. At the end of the 12 training sessions, all participants were individually reevaluated with tests (Figure 2).

#### Ethics statement

This project was approved by the Ethics Committee for Analysis of Research Projects of the Clinical Direction of Clinical Hospital, School of Medicine, University of São Paulo (Protocol: CAAE 538662016.0.00040.0068). All participants signed the Informed Consent Form in accordance with Resolution No. 466 (12/12/2012) of the National Health Council.

### Statistical analysis

The data’s statistical analysis was performed using the SPSS software, version 25.0. Initially, the sociodemographic data were compared at baseline between the EG and the CG, and the normal distribution of the continuous data was verified using the Shapiro-Wilks test. The Student’s t-test and the non-parametric Mann-Whitney test were used for independent samples, depending on the normalcy of the variables. For analysis of the categorical data and of the association between groups, the Fisher’s Exact test and the chi-square test were used. All tests were two-tailed with a required significance level of 0.05.

For the evaluation of differences between groups regarding pre- and post-training cognitive performance, Generalized Estimation Models (GEM) were used. According to Quasi Likelihood Under Independence Model Criterion (QIC) of model adjustment, gamma distribution was selected, with a logarithmic link matrix. For the evaluation of pairwise differences, the post-hoc Sidak-test was conducted. Post-hoc tests and the mean differences presented in them considered the variables in an isolated way, while β (beta) presented in Parameter Estimates was denotative of mean differences in the presence of the other variables, making it the most important.

Calculations of Estimated Marginal Means were conducted, including different levels of pairwise interaction and, in some cases, the reference level used was one which evidenced in the clearest way results from multivariate hypothesis tests (tests of model effects). For the score composed of memory, the z-score of the variables WM, VM and EM were calculated, and the arithmetic mean of the values (with TMT multiplied by -1) was added to the minimum value, in a way in which only positive values were used.

## RESULTS

Table 1 expresses the sociodemographic data of all 44 participants, 11 of whom were male, distributed 27% in the EG and 23% in the CG. A Student’s t-test was used to analyze the age range and there were no significant differences between the groups. The mean (M) and standard deviation (SD) of this sample age are 73±8 years for the EG and 73±7 years for the CG. Regarding the total sample, 68% had a spouse/partner and, of these, 77% were in the EG and 59% in the CG.

**Table 1.** Sociodemographic data of the sample.

	CG (n=22)	EG (n=22)	All (n=44)	**p*-*value**
Age, years	73±7	73±8	73±7	0.95
Male, n (%)	5 (23)	6 (27)	11 (25)	0.73
Marital status				0.20
Partner, n (%)	13 (59)	17 (77)	30 (68)	
No Partner, n (%)	9 (41)	5 (23)	14(32)	
Number of children	3±1	3±1	3±1	0.76
Education				0.45
≤4 years	7 (32)	3 (14)	10 (23)	
from 4-8 years	4 (18)	6 (27)	10 (23)	
from 8-12 years	4 (18)	3 (14)	7 (16)	
>12 years	7 (32)	10 (45)	17 (38)	
Physical activity				0.49
None, n (%)	7 (32)	7 (32)	14 (32)	
Low, n (%)	4 (18)	8 (36)	12 (28)	
Moderate, n (%)	6 (27)	3 (14)	9 (20)	
Intense, n (%)	5 (23)	4 (18)	9 (20)	

Values are mean±standard deviation or absolute and percentual number. CG: control group; EG: experimental group.

Based on the Mann-Whitney test, no significant difference in the number of offsprings between the groups was shown. The mean number of children in both groups was three per family. Regarding education, the total sample was divided into years of schooling, and 32% (n=14) of the participants studied for up to four years, 27% (n=12) from four to eight years; 21% between 8 and 12 years, and 21% (n=9) studied for more than 12 years. In terms of physical activity, 32% of the elderly participants do not practice any physical activity. Regarding gender, marital status, education, monthly income, and physical activities, a cross-tabulation showed homogeneity between the groups considering a significance level of 0.05.

### Cognitive training

Table 2 shows participants’ pre- and post-MT cognitive results regarding verbal fluency total letters (VFTL); verbal fluency total category (VFTC); NT score of visual ability - difficulty and speed ((NT-P) and NT score of visual ability - processing speed (NT-H)). Data showed the effect of time (p<0.05) on verbal fluency, displaying a significatively better performance in all participants. Nonetheless, this result was not seen considering the group factor (p=0.93) or the *interaction time group (p=0.75). An information processing analysis was carried out by the NT in the pre- and post-training phases. Results show improvement in the visual perception ability of the attentional stimulus (NT-H), statistically significant in time (p<0.05), although not significant considering the group factor (p=0.60), or the *time group interaction (p=0.19).

**Table 2.** Effects of memory training on verbal fluency and NeuroTracker over time, for the effect of group, time and group.

	CG		EG		p-value		
	T0	T1	T0	T1	G	T	G*T
VFTL	32±18	35±21	41±17	42±17	0.15	0.06	0.23
VFTC	29±9	34±11	33±10	39±11	0.09	<0.05	0.75
NT-P	2.75±0.74	3.15±1.14	3.03±3.03	3.11±0.90	0.97	0.32	0.88
NT-H	2.51±0.71	2.51±0.71	2.71±0.63	2.71±0.63	0.60	<0.05	0.19

Values are mean±standard deviation. CG: comparative group; EG: experimental group; T0: pre-training evaluation; T1: post-training evaluation; G: difference between groups; T: difference between times; G*T: interaction among groups and times; VFTL: verbal fluency total letters; VFTC: verbal fluency total category; NT-P: NeuroTracker score of visual ability - difficulty and speed; NT-H: NeuroTracker score of visual ability - processing speed; calculations based on the marginal means test by the generalized estimation models (p<0.05*) (significant interaction reference).

Table 3 presents the results of the cognitive training for group, time and age, as well as the results of the cognitive assessment for memory and attention concerning the interactions group*time and group*time*age.

**Table 3.** Analysis of the cognitive assessments for memory under the effect of group, age and the interaction between group*time and group*time*age.

	CG		EG		p-value				
	T0	T1	T0	T1	G	T	A	G*T	G*T*A
SRT	572±186	531±194	517±205	471±102	0.88	0.34	<0.05	<0.05	<0.05
CRT	725±138	666±144	664±166	668±141	0.55	0.86	0.27	<0.05	0.18
WM	1,185±248	1,266±542	1,295±457	1,352±627	0.59	0.72	<0.05	<0.05	<0.05
VM	86±13	88±10	90±11	89±17	0.99	0.31	0.62	0.58	0.78
EM	58±13	62±9	62±8	63±10	0.54	0.08	0.95	0.89	<0.05
MGR	1.32±0.55	1.53±0.64	1.59±0.60	1.67±0.67	0.65	<0.05	0.32	0.75	<0.05

Values are mean±standard deviation. CG: comparative group; EG: experimental group; T0: pre-training evaluation; T1: post-training evaluation; G: difference between groups; T: difference between times; A: corrected by age; G*T: interaction among groups and times; G*T*A: interaction among groups and times corrected by age; SRT: simple reaction time in millisecond; CRT: choice reaction time; WM: working memory; VM: verbal memory; EM: episodic memory; MGR: memory general result; calculations based on marginal means test by the generalized estimation models; (p<0.05) (meaningful interaction reference).

Data in Table 3 show that, in the analysis of SRT in milliseconds, considering participants’ age, no significant results were found in relation to the group (p=0.881) or time (p=0.340) separately. When analyzing data with age variable, or interaction group*time and group*time*age, in a singled out way, results were statistically significant (p<0.05). This suggests that the training carried out did in fact improve SRT, which indicates that the processing speed of information grew faster.

Regarding WM, group and time variables alone were not significant (p=0.592 and p=0.719), respectively. Nonetheless, data demonstrated a significant effect of age - both alone and in the interaction group*time and group*time*age (p<0.05*), which suggests an improvement in WM. In relation to the interaction group*time*age, significant differences (p<0.05) were observed, which shows improvement in EM performance. With respect to general memory results (GMR), there was an improvement in the results in relation to the effect of time and of group*time*age interaction (p<0.05), which suggests that the elderly benefited from combined training, presenting improved performance in memory tasks.

Table 4 presents the effect size of the test results (β value) using parameter estimation calculations (Wald chi-square test) for the isolated variables group and time, as well as for the interaction group*time. The Verbal Fluency Test shows improvement in relation to the categories mentioned, since, on average, the total sample presented a significant increase (chi-square (1)=17.13; p<0.001) with a post-intervention effect size of 0.15 points.

**Table 4.** Effect size results (β) of the cognitive and scale evaluations under the effect of group, time and the interaction group*time in estimates of specific parameters.

Total sample (n=44)					
Variables	Parameters	β	SD	chi-square	p-value*
VFTC	T0	-0.15	0.04	17.13	<0.001
NT-H	T0	-0.17	0.58	8.23	<0.01

VFTC: verbal fluency total category; NT-H: NeuroTracker score of visual ability - processing speed; T0: pre-training evaluation; β: effect size; SD: standard deviation); *Generalized Estimation Models; chi-square: Wald chi-square test.

Using the same measurement parameters, processing speed NT-H revealed good results, presenting significant increase in the total sample (chi-square (1)=8.23; p<0.01) and mean β=0.17 points in the post-intervention. These results indicate an increase in elderly participants’ visual processing speeds in the post intervention, compared with the memory and attention in the pre-training phase.

## DISCUSSION

The current study derived from an initial project aimed at implementing cognitive intervention programs in a Neuropsychology Unit of a Psychiatric Hospital in Brazil. Finally, the effectiveness of a memory training program was investigated, which consisted of 12 60-minute session for two groups of elderly with no cognitive complaints. Participants in the EG received MT and training associated with the software 3D-MOT, and in the CG, participants received only MT.

The idea of implementing a cognitive training program came from the 2015 Brazilian Census data, which showed that 14% of the 204.450.649 inhabitants of Brazil are 65 years old or over. In addition, according to the WHO^1^ predictions, in 2025 Brazil will rank the sixth highest country in the elderly population.

On the one hand, aging is known to have important effects on cognition, functionality, and quality of life as well as on the brain; however, this does not necessarily happen in association with a pathological process. On the other hand, aging may be affected by neurodegeneration such as Alzheimer disease (AD), which presents an extensive preclinical stage from 15 to 20 years before the emergence of clinical signs.^22^ ^,^ ^23^ Studies have shown, for example, that the neuropathological examination of elderly people who died with and without mild cognitive impairment, revealed pathological similarities to those with AD.^24^ According to these authors, this condition is characterized as an asymptomatic heterogeneous phase of AD that varies in the elderly population. In our perspective, two areas of study derive from these findings: one aimed at improving early diagnostic methods, such as biomarkers,^25^ and the other at stimulating programs or cognitive training for the elderly population,^3^ ^,^ ^4^ ^,^ ^25^ ^,^ ^26^ which is what was done in this study.

Cognitive training is designed based on the understanding that the human brain has a retention capacity potential for neuroplasticity until late adulthood.^8^ ^,^ ^27^ ^,^ ^28^ Thus, actions aimed to enhance cognitive functions or to delay their decline are promising.^28^ ^,^ ^29^ ^,^ ^30^ ^,^ ^31^ ^,^ ^32^ In this context, the literature has reported benefits of cognitive training and rehabilitation programs for the elderly population, establishing the need to invest in more specific designs of these intervention programs.^3^ ^,^ ^4^ ^,^ ^5^ ^,^ ^6^ ^,^ ^10^ ^,^ ^33^ ^,^ ^34^ Therefore, our data configures as a pilot study to verify the feasibility of cognitive training associated with 3D-MOT stimulus for aged participants without cognitive complaints, from a psychogeriatric outpatient clinic.

In the present study, it was possible to show that cognitive training programs can result in improvements in the cognitive performance of the elderly population. This was demonstrated since the total sample showed significant improvements in the post-intervention phase in evaluated functions such as attention, memory, information processing speed, and learning. These results converge with the literature which, although not specifically for the elderly population, also report efficacy for cognition.

Carvalho et al.,^35^ conducted a study with 57 aged participants (26 in the CG and 31 in the EG). The participants in the EG were evaluated, followed by five episodic memory sessions (twice a week, 60 minutes each session, in which they were instructed to categorize supermarket and figures lists) and then were reevaluated. The CG received an abbreviated version of the training with pre- and post-evaluation. The results indicated that MT promoted a significant improvement in the performance of an episodic memory task and greater strategy use trained in the EG.

Another study with 76 aged participants without cognitive complaints or neurodegeneration, aimed to evaluate the cognitive effects of attention, memory, and executive functions training in a CG and EG with 38 participants in each group. The participants of the EG received 12 sessions (90 minutes each) of attention, memory, and executive functions training and the CG performed activities aimed at cognitive improvement.^35^ Results showed better performance of the executive functions in the post-test of the EG when compared to the CG.^36^ Cujzek and Vranic^37^ performed a videogame intervention in 29 aged participants, where aspects of cognitive function were evaluated (vigilance, operational memory, inhibition, and reasoning before and after six weeks of practice). The difference between the groups was in the content of extended videogame practice, the EG used a cognitively complex and computerized card game and the CG only played a dice game. The intervention lasted for four months and the results showed improvement in both groups, except for reasoning, in which the EG presented better results. These results suggest that the improvements were related to the program’s complexity and that cognitive stimulating activity is a valid training procedure for the elderly population. According to the authors, another important item in determining training effectiveness is familiarity with computers.

The MT of the current study was intentionally structured in 12 60-minute sessions, organized twice a week. The composition of the mnemonic strategies was designed to determine the effectiveness in the daily lives of the aged participants and, consequently, an improvement in the quality of life as reported in the literature.^10^ ^,^ ^12^ ^,^ ^33^ ^,^ ^34^ ^,^ ^35^ ^,^ ^38^

In general, specifically regarding MT, participants of the EG and CG obtained cognitive improvements in the evaluated aspects and it was evident that the use of strategies - proven through the numbers of categories evoked in the post-training - showed that the mnemonic process significantly enhanced the memory capacity of the participants. In addition, the results showed better scores in episodic and working memory and in the general memory score as well as in attention and in information processing speed, thus reflecting an improvement in the cognitive performance of this population. Within this scope, these results corroborate the literature regarding the effects of memory training.^12^ ^,^ ^33^ ^,^ ^34^ ^,^ ^35^ ^,^ ^38^ ^,^ ^39^ ^,^ ^40^ ^,^ ^41^ ^,^ ^42^

The results of this study also suggest that the use of the MT program in combination with the attentional training through 3D-MOT was innovative and demonstrated positive effects on attention, working memory, and visual information processing speed in the aged participants in the EG. It is noteworthy that the 3D-MOT has already proven to be an important technological resource for cognition in athletes, as it stimulates the peripheral vision resulting in the improvement of information processing speed.^17^ Therefore, with the association of MT and NT, this combination was proven to be an important resource with strong potential to promote cognitive improvement in the aged population, especially in regard to memory and information processing speed. It was observed that participants who had sessions of MT associated with NT were faster to perform the same task after the intervention, a result that converges with the literature, however from studies with different aged populations.^26^ ^,^ ^43^ As an example, it is worth mentioning the study by Harenberg and colleagues,^44^ which evaluated a sample of 29 aspiring surgeons in NT training, which expressed improvements in visual tracking skills and information processing speed.

Parsons et al.,^14^ in a study with 20 college students using the NT, showed that 10 cognitive training sessions and functional brain imaging, resulted in an improvement of attention, visual information processing speed, and WM, in addition to presenting quantifiable changes in neuroelectric brain function at rest. In another study, individuals aged 64 to 73 years were trained in a speed task in comparison to the CG; results showed that training can be a good generic process to help certain observers cope with socially relevant dynamic scenes.^3^

In this study it was possible to demonstrate that the use of NT as a tool to stimulate attention brought greater benefit to the EG, confirming our hypothesis that the MT associated with the attentional stimulus (NT) would be more effective than only MT, as it results in faster information processing speed. The MT was designed to last for a longer period than those reported in the literature, which describe the effectiveness of shorter interventions, some consisting of five sessions only.^35^

It should be noted that this article resulted from a pilot study, whose objective was to implement a cognitive training program for the elderly. And, although we are also aware that the literature discusses the effectiveness of cognitive training, the results, even with low generalization power, are encouraging, but it is fully understood that surpassing limitations such as sample size and the inclusion of elderly groups with cognitive complaints for comparison will certainly contribute to a better expressiveness of these results. Another limitation found in this pilot study was the lack of a six-month follow-up assessment. If these limitations were addressed, more consistent data regarding the effect of cognitive training in the elderly population could possibly be obtained. Future studies should focus on eliminating these limitations.

Despite being a pilot and with low generalization power, this study showed that the combination of memory training and 3D-MOT contributed to a better cognitive performance in the experimental group. Thus, we understand that the results of the present study encourage the continuity of research and the development of combined cognitive interventions for the elderly population with and without cognitive deficits.

## References

[1] World Health Organization (2017). Centre for Health Development.

[2] Marioni RE, Marioni RE, Valenzuela MJ, van den Hout A, Brayne C, Matthews FE (2012). Active cognitive lifestyle is associated with positive cognitive
health transitions and compression of morbidity from age
sixty-five. PLoS ONE.

[3] Legault I, Allard R, Faubert J (2013). Healthy older observers show equivalent perceptual-cognitive
training benefits to young adults for multiple object
tracking. Front Psychol.

[4] Arnemann KL, Chen AJ, Novakovic-Agopian T, Gratton C, Normura EM, D’Esposito M (2015). Functional brain network modularity predicts response to
cognitive training after brain injury. Neurology.

[5] Rodakowski J, Saghafi E, Butters MA, Skidmore ER (2015). Non-pharmacological interventions for adults with mild cognitive
impairment and early stage dementia: An updated scoping
review. Molecular Aspects of Medicine.

[6] Giuli C, Fattoretti P, Gagliardi C, Mocchegiani E, Venarucci D, Balietti M (2017). My Mind Project: the effects of cognitive training for
elderly-the study protocol of a prospective randomized intervention
study. Aging Clin Exp Res.

[7] Sohlberg MM, Mateer CA (2001). Cognitive rehabilitation: an integrative neuropsychological
approach.

[8] Karbach J, Schubert T (2013). Training-induced cognitive and neural plasticity. Front Hum Neurosci.

[9] Sherman DS, Mauser J, Nuno M, Sherzai D (2017). The efficacy of cognitive intervention in mild cognitive
impairment (mci): a meta-analysis of outcomes on neuropsychological
measures. Neuropsychol Rev.

[10] Bender AR Naveh-Benjamin M, Raz N (2010). Associative deficit in recognition memory in a lifespan sample of
healthy adults. Psychol Aging.

[11] Lampit A, Hallock H, Valenzuela M (2014). Computerized cognitive training in cognitively healthy older
adults: a systematic review and meta-analysis of effect
modifiers. PLoS Med.

[12] Assed MM, Carvalho MKHV, Rocca CCA, Serafim AP (2016). Memory training and benefits for quality of life in the elderly:
A case report. Dement Neuropsychol.

[13] Faubert J, Sidebottom L (2012). Perceptual-cognitive training of athletes. J Clin Sport Psychol.

[14] Parsons B, Magill T, Bouche A, Zhang M, Zogbo K, Bérubé S (2014). Enhancing cognitive function using perceptual-cognitive
training. Clin EEG Neurosci.

[15] Faubert J (2013). Professional athletes have extraordinary skills for rapidly
learning complex and neutral dynamic visual scenes. Sci Rep.

[16] Mangine GT, Hoffman JR, Wells AJ, Gonzalez AM, Rogowski JP, Townsend JR (2014). Visual tracking speed is related to basketball-specific measures
of performance in NBA players. J Strength Cond Res.

[17] Vartanian O, Coady L, Blackler K (2016). 3D multiple object tracking boosts working memory span:
Implications for cognitive training in military populations. Milit Psychol.

[18] Folstein MF, Folstein SE, McHugh PR (1975). Mini-mental state: A practical method for grading the cognitive
state of patients for the clinician. J Psychiatr Res.

[19] Wechsler D (2014). Wechsler abbreviated scale of intelligence.

[20] Rodrigues AB, Yamashita ET, Chiappetta ALML (2008). Verbal fluency test in adult and elderly: verification of verbal
learning. Rev CEFAC.

[21] Moffat N, Wilson BA, Moffat N (1992). Strategies of memory therapy. Clinical Management of Memory Problems.

[22] Sperling RA, Aisen PS, Beckett LA, Bennett DA, Craft S, Fagan AM (2011). Toward defining the preclinical stages of Alzheimer's disease:
recommendations from the National Institute on Aging-Alzheimer's Association
workgroups on diagnostic guidelines for Alzheimer's disease. Alzheimers Dement.

[23] Sperling RA, Karlawish J, Johnson KA (2013). Preclinical Alzheimer disease-the challenges
ahead. Nat Rev Neurol.

[24] Morris RG, Worsley C, Mattewa D (2000). Neuropsychological assessment in older people: old principles and
new directions. Adv Psychiatr Treat.

[25] Counts SE, Ikonomovic MD, Mercado N, Vega IE, Mufson EJ (2017). Biomarkers for the early detection and progression of Alzheimer's
disease. Neurotherapeutics.

[26] Chamoun M, Huppé-Gourgues F, Legault I, Rosa-Neto P, Dumbrava D, Faubert J (2017). Cholinergic potentiation improves perceptual-cognitive training
of healthy young adults in three dimensional multiple object
tracking. Front Hum Neurosci.

[27] Melby-Lervåg M, Hulme C (2013). Is working memory training effective? A meta-analytic
review. Dev Psychol.

[28] Leung NT, Tam HM, Chu LW, Kwok TCY, Chan F, Lam LCW (2015). Neural plastic effects of cognitive training on aging
brain. Neural Plast.

[29] Scholz J, Klein MC, Behrens TE, Johansen-Berg H (2009). Training induces changes in white-matter
architecture. Nat Neurosci.

[30] May A (2011). Experience-dependent structural plasticity in the adult human
brain. Trends Cogn Sci.

[31] Brehmer Y, Westerberg H, Bäckman L (2012). Working-memory training in younger and older adults: training
gains, transfer, and maintenance. Front Hum Neurosci.

[32] Han K, Chapman SB, Krawczyk DC (2018). Neuroplasticity of cognitive control networks following cognitive
training for chronic traumatic brain injury. Neuroimage Clin.

[33] Netto MT, Fonseca RP, Landeira-Fernandez J (2012). Memory rehabilitation of elderly adults with mnemonic complaints
and depressive symptoms: a pilot study. Est Psicol (Natal).

[34] Silva TBL, Oliveira ACV, Paulo DLV, Yassuda MS (2011). Cognitive training for elderly adults based on categorization
strategies and calculations similar to everyday tasks. Rev Bras Geriatr Gerontol.

[35] Carvalho FC, Neri A, Yassuda MS (2010). Episodic memory training with emphasis on categorization for
older adults without dementia and depression. Psicol Reflex Crít.

[36] Irigaray TQ, Filho IG, Schneider RH (2010). Effects of an attention, memory and executive functions training
on the cognition of healthy elderly people. Psicol Reflex Crít.

[37] Cujzek M, Vranic A (2017). Computerized tabletop games as a form of a video game training
for old-old. Aging Neuropsychol Cogn.

[38] Schultheisz TSV, Aquino RR, Alves ABF, Radl ALM, Serafim AP (2018). Effect of cognitive stimulation workshops on the self-esteem and
cognition of the elderly A pilot project. Dement Neuropsychol.

[39] Souza JN, Chaves EC (2006). The effect of memory stimulation practices as a therapeutic
method on healthy elders. Rev Esc Enferm USP.

[40] Willis SL, Tennstedt SL, Marsiske M, Ball K, Elias J, Koepke KM (2006). Long-term effects of cognitive training on everyday functional
outcomes in older adults. JAMA.

[41] Levine B, Stuss DT, Winocur G, Binns MA, Fahy L, Mandic M (2007). Cognitive rehabilitation in the elderly: Effects on strategic
behavior in relation to goal management. J Intern Neuropsychol Soci.

[42] Gross AL, Rebok GW (2011). Memory training and strategy use in older adults: results from
the ACTIVE study. Psychol Aging.

[43] Fragala MS, Beyer KS, Jajtner AR, Townsend JR, Pruna GJ, Boone CH (2014). Resistance exercise may improve spatial awareness and visual
reaction in older adults. J Strength Cond Res.

[44] Harenberg S, McCaffrey R, Butz M, Post D, Howlet J, Dorsch K (2016). Can multiple object tracking predict laparoscopic surgical
skills?. J Surg Educ.

